# Strategies to improve the accuracy and reduce costs of genomic prediction in aquaculture species

**DOI:** 10.1111/eva.13262

**Published:** 2021-07-17

**Authors:** Hailiang Song, Hongxia Hu

**Affiliations:** ^1^ Beijing Fisheries Research Institute & Beijing Key Laboratory of Fishery Biotechnology Beijing China

**Keywords:** aquaculture species, genomic selection, methods, reduce costs

## Abstract

Genomic selection (GS) has great potential to increase genetic gain in aquaculture breeding; however, its implementation is hindered owing to high genotyping cost and the large number of individuals to genotype. This study investigated the efficiency of genomic prediction in four aquaculture species. In total, 749 to 1481 individuals with records for disease resistance and growth traits were genotyped using SNP arrays ranging from 12K to 40K. We compared the prediction accuracies and bias of breeding values obtained from BLUP, genomic BLUP (GBLUP), Bayesian mixture (BayesR), weighted GBLUP (WGBLUP), and genomic feature BLUP (GFBLUP). For GFBLUP, the genomic feature matrix was constructed based on prior information from genome‐wide association studies. Fivefold cross‐validation was performed with 20 replicates. Moreover, to reduce the cost of GS, we reduced the SNP density based on linkage disequilibrium as well as the reference population size. The results showed that the methods with marker information produced more accurate predictions than the pedigree‐based BLUP method. For the genomic model, BayesR performed prediction with a similar or higher accuracy compared to GBLUP. For the four traits, WGBLUP yielded an average of 1.5% higher accuracy than GBLUP. However, the accuracy of genomic prediction decreased by an average of 6.2% for GFBLUP compared to GBLUP. When the density of SNP panels was reduced to 3K, which was sufficient to obtain accuracies similar to those using the whole dataset in the four species, the cost of GS was estimated to be 50% lower than that of genotyping all animals with high‐density panels. In addition, when the reference population size was reduced by 10%, evenly from full‐sib family, the accuracy of genomic prediction was almost unchanged, and the cost reduction was 8% in the four populations. Our results have important implications for translating the benefits of GS to most aquaculture species.

## 1 INTRODUCTION

Genomic selection (GS) has developed rapidly since its proposal in 2001 (Meuwissen et al., 2001). It has dominated research and development and has brought revolutionary changes to animal and plant breeding. In GS, quantitative trait loci (QTL) are presumed to be in linkage disequilibrium (LD) with at least one of the genotyped markers that are used to estimate the level of genetic similarity between individuals and explain the genetic variance for the trait. Compared with pedigree‐based prediction of breeding values, GS can be performed as soon as DNA is available, which allows for accurate selection early in life. Theory and breeding practices indicate that the accuracy of GS is higher than that of traditional breeding methods, which can speed up breeding progress and improve breeding efficiency (García‐Ruiz et al., 2016; Goddard & Hayes, 2007).

Genomic selection in aquaculture species was first studied in Atlantic salmon, and its application was made possible by the development of the first high‐density (HD) SNP arrays and demonstration of their utility to accurately predict breeding values in a typical salmon breeding program (Houston et al., 2014; Odegard et al., 2014). With the increase in fish whole‐genome sequencing and the reduction in the cost of resequencing, research on GS in aquaculture species has gradually developed. GS has been applied to complex traits of several important aquaculture species (Houston et al., 2020; Zenger et al., 2019), including Atlantic salmon (*Salmo salar*) (Tsai et al., 2015, 2016; Tsairidou et al., 2020), rainbow trout (*Oncorhynchus mykiss*) (D'Ambrosio et al., 2020; Vallejo et al., 2016, 2018), large yellow croaker (*Larimichthys crocea*) (Zhao et al., 2021), Nile tilapia (*Oreochromis niloticus*) (Joshi et al., 2020; Penaloza et al., 2020), *Penaeus vannamei* (*Litopenaeus vannamei*) (Wang et al., 2017), Japanese flounder (*Paralichthys olivaceus*) (Lu et al., 2020), and rock bream (*Oplegnathus* *fasciatus*) (Gong et al., 2021).

Most GS studies in aquaculture species use the best linear unbiased prediction (genomic BLUP, GBLUP) method based on a genomic relationship matrix that estimates the genomic estimated breeding value (GEBV; Zenger et al., 2019). Bayesian models have also been tested in several species, but compared with the simpler GBLUP method, for Bayesian models, the prediction accuracy is only slightly higher or not significantly different (Zenger et al., 2019) and needs more computing demands, and the performance depends on the underlying genetic architecture of the traits. Moreover, incorporating preselected potential causal markers into the GS model is an effective way to improve the accuracy of prediction. For example, Lu et al. (2020) used a genome‐wide association study (GWAS) to preselect sequencing data, as well as single‐step GBLUP (ssGBLUP), weighted BayesB, and BayesB to predict the breeding value of Japanese flounder against *Edwardsiella* infection, and found that preselecting SNPs improved the accuracy of genomic prediction. Dong et al. (2016) reported that the accuracy of genomic prediction for two growth traits could be improved when SNPs were preselected based on the largest absolute effects of SNPs in large yellow croaker; additionally, Yoshida and Yáñez (2021) reported that the accuracy of genomic prediction can be improved using preselected variants from GWASs for growth under chronic thermal stress in rainbow trout. Thus, the use of preselected SNPs could be an attractive approach for increasing accuracy.

Owing to the high fecundity of aquatic animals, the application of GS in aquaculture species is very expensive because of the large number of selected candidates and test‐sibs for genotyping. Genotyping a large number of animals with an HD SNP panel is not realistic for all but the largest aquaculture breeding companies. Thus, it is important to develop cost‐effective GS strategies for aquaculture species. Several strategies have been proposed to reduce the cost of genotyping for GS in aquaculture via low‐density SNP panels (Kriaridou et al., 2020), low‐coverage sequencing (Zhang et al., 2021), and the use of genotyping strategies, including imputation from low‐to‐high‐density SNPs (Tsai et al., 2017). Genotype‐by‐sequencing technologies are also likely to help reduce costs in aquaculture; Vallejo et al. (2016) compared the prediction accuracy of RAD sequencing (RAD‐seq) and SNP chips on bacterial cold‐water disease resistance of rainbow trout and found that although the marker density of SNP chip was higher (approximately 40K SNP–10K RAD‐seq), the selection accuracy of the two technologies was similar. Reducing the cost of GS is critical for implementing GS in most aquaculture breeding programs.

The objectives of this study were to (1) evaluate the accuracy of GS for different traits in four aquaculture species using GBLUP and Bayesian mixture (BayesR) methods; (2) explore strategies to improve the accuracy of genomic prediction using weighted GBLUP (WGBLUP) and genomic feature BLUP (GFBLUP) with preselected SNPs; and (3) explore strategies to reduce the cost of GS.

## 2 MATERIALS AND METHODS

### 2.1 Population and phenotypes

The phenotypes were obtained from four previously published studies of four different species. Briefly, (1) Atlantic salmon (*S. salar*) challenged with amoebic gill disease were phenotyped for mean gill score (mean of the left gill and right fill scores, a continuous trait), and a subjective gill lesion score of the order of severity ranging from 0 to 5 was recorded for both gills. The challenged fish belonged to 84 different full‐sib families, with 1–39 fish in each family (Robledo et al., 2018); (2) common carp (*Cyprinus carpio*), from four factorial crosses of five females × ten males (20 females and 40 males in total), were measured for body weight. These fish belonged to 195 full‐sib families, with 1–21 fish in each family (Palaiokostas et al., 2018); (3) sea bream (*Sparus aurata*), originating from a factorial cross between 67 broodfish (32 males and 35 females), were challenged by 30‐min immersion with 1 × 10^5^ CFU *Photobacterium damselae* (causative agent of pasteurellosis) and the number of days to death was recorded. These fish belonged to 73 full‐sib families, with 2–144 fish in each family (Palaiokostas et al., 2016); and (4) rainbow trout (*O*. *mykiss*) belonged to 58 full‐sib families generated from 58 females and 20 males of rainbow trout from the 2014 year class, with 10–18 fish in each family. These fish were challenged with infectious pancreatic necrosis, and the number of days to death was recorded (Rodriguez et al., 2019). A summary of the dataset is provided in Table 1.

**TABLE 1:** Descriptive statistics for four traits of four species, including the number of observations and full‐sib families

Species	Trait	N‐obs	Full‐sib families	Mean (SD)
Atlantic salmon	Mean gill score	1481	84	2.79 (0.85)
Common carp	Body weight	1214	195	16.32 (4.58)
Sea bream	Number of days to death	777	73	10.34 (4.09)
Rainbow trout	Number of days to death	749	58	51.47 (13.98)

Abbreviations: N‐obs, number of observations; SD, standard deviation.

### 2.2 Genotype data and imputation

(1) Atlantic salmon, a total of 1481 Atlantic salmon were genotyped using an Illumina combined species of Atlantic salmon and rainbow trout 17K SNP array (17156 SNPs), designed from a subset of SNPs from a higher density array (Houston et al., 2014); (2) common carp, 1214 fish were genotyped using RAD‐seq for ~12K SNPs; (3) sea bream, 777 fish were genotyped using 2b‐RAD‐seq for ~12K SNPs; and (4) rainbow trout, 749 fish were genotyped using a 57K Affymetrix Axiom SNP array developed by Palti et al. (2015).

Imputation for missing genotypes of SNPs with known chromosomal positions was performed using Beagle4.1 (Browning & Browning, 2009). The SNPs were filtered using the following quality control criteria from the imputed dataset: minor allele frequency (MAF) <0.01, SNP call rates <0.90, and genotype frequency deviating from Hardy–Weinberg equilibrium (HWE) with a *p*‐value <10^−7^. Individuals with call rates <0.90 were also excluded. Quality control was performed using the Plink software package (v1.90; Chang et al., 2015). After quality control, all genotyped individuals remained, and the SNPs ultimately used were 11,068, 12,311, 12,050, and 40,143 for Atlantic salmon, common carp, sea bream, and rainbow trout, respectively.

### 2.3 Statistical models

Four methods, GBLUP, BayesR, WGBLUP, and GFBLUP, were implemented to predict GEBV for each genotyped individual.

#### 2.3.1 GBLUP

The GBLUP (VanRaden, 2008) model based on the genomic relationship matrix was used to predict the GEBV for all genotyped individuals.

y=Xb+Lg+e,

where **y** is the vector of observed phenotypic values; **b** is the vector of fixed effects; **g** is the vector of additive genetic effects, following a normal distribution of $N(0,\;G\sigma_{a}^{2}),$ where $\sigma_{a}^{2}$ is the additive genetic variance, and **G** is the genomic relationship matrix (VanRaden, 2008). The **G** matrix was constructed using all markers as $G={\mathrm{ZZ}}^{\prime }/\sum 2p_{j}\left(1‐p_{j}\right)$, where p_j_ is the reference allele frequency of A_2_ for genotypes A_1_A_1_, A_1_A_2_, and A_2_A_2_ at locus j; and **Z** is a centered genotyped matrix where genotypes are subtracted 2 * reference allele frequency. In this study, the allele frequencies p_j_ were estimated from current marker data. **X** and **L** are the incidence matrices that relate **b** and **g** to **y**, respectively; **e** is the vector of random errors with distribution of N(0, **I** $\sigma_{e}^{2}$), where $\sigma_{e}^{2}$ is the residual variance, and **I** is the identity matrix. The different fixed effects included in the model for different species were overall mean, collection date (three levels), and tank (two levels) in Atlantic salmon; overall mean and factorial‐cross group (4 levels) for common carp; overall mean in sea bream; and overall mean and tagging weight as a covariate in rainbow trout.

#### 2.3.2 BayesR

The BayesR model (Erbe et al., 2012) was used to predict the GEBV for each individual. For BayesR, all SNP effects were estimated based on the reference population, and the GEBV of a genotyped individual was calculated as the sum of all SNP effects according to the SNP genotypes. The following model was used to estimate the effects of all the SNPs simultaneously:

y=Xb+Wu+Lv+e,

where **y**, **X**, **L**, **b**, and **e** are the same as in the GBLUP model, **W** is the (n × m) design matrix allocating records to the marker effects; and **u** is an (m × 1) vector of SNP effects assumed to be normally distributed $u_{i}∼N\left(0,\sigma_{i}^{2}\right).$ The variance of the *i*th SNP effect had four possible values: $\sigma_{1}^{2}=0$, $\sigma_{2}^{2}=0.0001\sigma_{g}^{2}$, $\sigma_{3}^{2}=0.001\sigma_{g}^{2}$, $\sigma_{4}^{2}=0.01\sigma_{g}^{2}$, where $\sigma_{g}^{2}$ is the total genetic variance; **v** is a vector of random residual polygenic effects with a normal distribution *g* ~ N(0, **A** $\sigma_{a}^{2}$), where $\sigma_{a}^{2}$ is the polygenic variance, and **A** is the pedigree relationship matrix. The Markov chain Monte Carlo (MCMC) was run for 50,000, and the first 10,000 cycles were discarded as burn‐in, and every 10th sample of the remaining 40,000 iterations was saved for estimating SNP effects and the variance components.

#### 2.3.3 WGBLUP

The WGBLUP (Wang et al., 2012) has the same model as GBLUP, except that **G** is the weighted genomic relationship matrix. The iterative steps in WGBLUP are as follows.

1. Set t = 0, **D**_(t)_ = **I**, and $G_{\left(t\right)}={\mathrm{ZD}}_{\left(t\right)}Z^{\prime }\frac{1}{\sum 2p_{j}\left(1‐p_{j}\right)}$, where t is the iteration number and **Z**, **I**, and p_j_ are the same as those described in the GBLUP method.
2. Construct matrix $G_{\left(t\right)}^{*}=0.95*G_{\left(t\right)}+0.05*I$ (VanRaden, 2008);
3. Calculate GEBV ($\hat{g}$) using GBLUP;
4. Calculate SNP effects as $\hat{u}=\frac{1}{\sum 2p_{j}\left(1‐p_{j}\right)}D_{\left(t\right)}Z^{\prime }{\left(G_{\left(t\right)}^{*}\right)}^{‐1}\hat{g}$;
5. Calculate SNP weight matrix $D_{\left(t+1\right)}^{*}$ as $d_{\text{jj(t}+1)}^{*}={\hat{u}}_{j}^{2}2p_{j}\left(1‐p_{j}\right)$;
6. Normalize matrix **D**_(t+1)_ as $D_{\left(t+1\right)}=\frac{{\text{tr(D}}_{\left(0\right)})}{\text{tr(}D_{\left(t+1\right)}^{*})}D_{\left(t+1\right)}^{*}$;
7. Construct the matrix $G_{\left(t+1\right)}^{*}=0.95*{\mathrm{ZD}}_{\left(t+1\right)}Z^{\prime }\frac{1}{\sum 2p_{j}\left(1‐p_{j}\right)}+0.05*I$, t = t+1;
8. Go back to step (3) when t is less than or equal to 4. The result from the third iteration was used for GS analysis, as there was no difference between the results of the third and fourth iterations in this study.

#### 2.3.4 GFBLUP

The GFBLUP (Edwards et al., 2016) model, which uses prior information about genomic features, is based on a linear mixed model with two random genomic effects:

y=Xb+Lf+Lr+e,

where **y**, **X**, **b**, **L**, and **e** are the same as in the GBLUP model; **f** is the vector of genomic values captured by genetic markers associated with a genomic feature of interest, following a normal distribution of $N(0,G_{f}\sigma_{f}^{2})$; **r** is the vector of genomic effects captured by the remaining set of genetic markers, following a normal distribution $N(0,G_{r}\sigma_{r}^{2})$; and **L** is an incidence matrix that links **f** and **r** to **y**. Matrices **G_f_** and **G_r_** were constructed in the same way as **G**, but using only the genetic marker set defined by a genomic feature, as described below, and the remaining markers, respectively.

A GWAS was used to define genetic markers that formed different classes of genomic features used in the GFBLUP model analyses. The statistical model used was as follows:

y=Xb+Lg+xc+e,

where **y**, **X**, **b**, **L**, and **e** are the same as in the GBLUP model, c is the additive effect of the variant to be tested for association, and **x** is the vector of the variant's genotype indicator variable coded as 0, 1, or 2. The analysis was based only on the reference data. A *p*‐value of 0.05 was used to assess the statistical significance of the effect of individual SNPs. When an SNP was significantly associated with phenotypes based on the prespecified significance cutoff level, the corresponding SNP was considered to define a genomic feature.

WGBLUP was implemented using the blupf90 software package (Misztal et al., 2014), and the DMUAI procedure, implemented in the DMU software (Madsen et al., 2018), was used for GBLUP and GFBLUP analyses.

### 2.4 Reduction in SNP density and reference population size

When two SNPs are in high LD, their genotypic information is redundant, and only one is necessary to represent the variation in neighboring regions. Moreover, animals related to the full‐sib family may partly explain the same part of the variation. Thus, reducing SNP density based on LD and the size of the full‐sib family may have little effect on genomic prediction accuracy. In this study, SNP panel genotypes were pruned based on LD to reduce the SNP density, and different SNP densities of 10K, 5K, 3K, 1K, and 0.5K were used to assess prediction accuracy. Furthermore, reduced reference population sizes were tested by evenly sampling a ratio of 10%, 20%, 30%, 40%, and 50% of the reference population from each full‐sib family to assess the accuracy of genomic prediction. To ensure that the reference population covers the whole family, the reduced number of individuals were only selected from families whose family size is larger than the reduced number. Thus, the number of families was kept the same but family sizes were reduced. Furthermore, we evaluated the impact of reduced SNP density and reduced population size on prediction accuracy by using subsets of data for the GBLUP.

### 2.5 Evaluation of the accuracy of genomic prediction

In this study, the accuracy and bias of prediction were obtained through a fivefold cross‐validation (CV). The genotyped individuals were randomly split into five folds, phenotypes from onefold (validation population) were removed from the dataset, and the remaining folds (reference population) were used to predict the GEBV in the validation population. This fivefold CV was replicated 20 times, resulting in 20 average accuracies of genomic prediction. The validation population was the same in each replicate of fivefold CV for all the four methods, GBLUP, BayesR, WGBLUP, and GFBLUP, and for assessing the impact of reducing SNP density and reference population size. The accuracy of genomic prediction was evaluated as r (y, GEBV)/$\sqrt{h^{2}}$, the correlation between GEBVs of the validation population and phenotypic values y divided by the square root of heritability *h* ^2^, as listed in Table 2. In addition, b (y, GEBV), the regression of y on GEBVs, was calculated to assess the possible bias of predictions, which is equal to the absolute value of the regression coefficient minus 1.

**TABLE 2:** Accuracy and bias of prediction using different models through 20 replicates of fivefold cross‐validation in four populations

Species (heritability (SE)^a^)	Method	Accuracy	Regression coefficient
Atlantic salmon (0.25 (0.06))	BLUP	0.510 (0.106)	1.012 (0.281)
	GBLUP	0.615 (0.101)	1.019 (0.224)
	BayesR	0.611 (0.102)	1.054 (0.259)
	WGBLUP	0.627 (0.101)	0.900 (0.243)
	GFBLUP	0.560 (0.102)	0.513 (0.107)
Common carp (0.26 (0.06))	BLUP	0.591 (0.113)	0.980 (0.239)
	GBLUP	0.635 (0.125)	1.046 (0.241)
	BayesR	0.747 (0.124)	0.994 (0.200)
	WGBLUP	0.657 (0.114)	0.892 (0.259)
	GFBLUP	0.540 (0.129)	0.478 (0.119)
Sea bream (0.12 (0.06))	BLUP	0.462 (0.197)	1.243 (0.816)
	GBLUP	0.625 (0.204)	1.153 (0.586)
	BayesR	0.643 (0.206)	1.906 (1.017)
	WGBLUP	0.636 (0.206)	0.960 (0.524)
	GFBLUP	0.574 (0.193)	0.382 (0.143)
Rainbow trout (0.50 (0.06)*)	BLUP	NA	NA
	GBLUP	0.816 (0.079)	0.992 (0.126)
	BayesR	0.829 (0.072)	0.978 (0.118)
	WGBLUP	0.831 (0.076)	0.929 (0.123)
	GFBLUP	0.771 (0.082)	0.730 (0.087)

Note

Standard deviations in brackets for accuracy and regression coefficient. NA: The BLUP method was not available because of the lack of pedigree in the rainbow trout population.

Abbreviations: BayesR, Bayesian mixture model; BLUP, BLUP method based on pedigree; GBLUP, genomic BLUP; GFBLUP, genomic feature BLUP with GWAS *p*‐value of 0.05 as genomic feature; WGBLUP, weighted GBLUP.

^a^ Heritability (standard error, SE) estimated using pedigree relationship information, except for rainbow trout (* heritability estimated using genomic relationship information).

### 2.6 Cost evaluation

We evaluated direct savings when genotyping a proportion of animals using different density SNP panels. Costs were calculated for four different species in this study. The genotyping cost was calculated assuming prices of $60, $50, $30, $25, $20, and $10 per sample for HD (from 12K to 40K for four species), 10K, 5K, 3K, 1K, and 0.5K, respectively. Here, we did not assume a price reduction when more animals were genotyped, as the population was not large in the four species in this study.

## 3 RESULTS

### 3.1 Descriptive statistics and genetic parameters

The descriptive statistics and genetic parameters for the analyzed traits of the four species are presented in Tables 1 and 2. In the Atlantic salmon population, the average mean gill score was 2.79, with a standard deviation of 0.85, and the heritability estimate for the mean gill score was 0.25, with a standard error of 0.06. In the common carp population, body weight with mean and standard deviation of 16.32 and 4.58, and the heritability was 0.26, with a standard error of 0.06. In sea bream and rainbow trout populations, the numbers of days to death with mean (standard deviation) were 10.34 (4.09) and 51.47 (13.98), respectively, and the heritability (standard error) estimates for the number of days to death were 0.12 (0.06) and 0.50 (0.06), respectively. It should be noted that the pedigree was not available for the rainbow trout population, and heritability was estimated using genomic relationship information.

### 3.2 Accuracy and bias of the GBLUP and BayesR methods

Table 2 presents the accuracy and bias of genomic prediction from 20 replicates of fivefold CV in four fish populations by applying the BLUP, GBLUP, BayesR, WGBLUP, and GFBLUP methods. As shown in Table 2, the four methods with marker information generally provided higher accuracies of genomic prediction than the traditional BLUP method, except that a slightly lower prediction accuracy was obtained for the GFBLUP method in the common carp population, and the BLUP method was not available for the rainbow trout population. In the Atlantic salmon population, similar prediction accuracies were obtained for the GBLUP and BayesR methods; the prediction accuracies obtained with GBLUP and BayesR were 0.615 and 0.611 and on average yielded 10.5% and 10.1% higher accuracies than BLUP, respectively. In the common carp population, the prediction accuracy obtained using GBLUP was 0.635. However, BayesR yielded 11.2% higher accuracy than GBLUP. Similarly, in sea bream and common carp populations, BayesR yielded 1.8% and 1.3% higher accuracies than GBLUP, respectively, which indicates that the prediction accuracy of the BayesR method depends on the genetic architecture of the traits. For bias of genomic prediction, as shown in Table 2, the regression coefficients of GBLUP and BayesR were close to 1, except for a large regression coefficient of 1.906 for BayesR in sea bream.

### 3.3 Methods to improve the accuracy of genomic prediction

Two methods, WGBLUP and GFBLUP, were performed using 20 replicates of fivefold CV to improve the accuracy of genomic prediction. As shown in Table 2, WGBLUP produced a higher prediction accuracy than GBLUP in four populations, and improvements were on average 1.5% for the four traits. The highest increase was 2.2% for body weight in the common carp population with WGBLUP compared to the GBLUP method. To evaluate the effect of including prior information, GFBLUP methods with GWAS information were compared with other methods. However, the GFBLUP model with *p *= 0.05, when using GWAS prior information, did not yield higher prediction accuracies than GBLUP and even produced the lowest prediction accuracies compared with other methods with marker information. The accuracy of genomic prediction decreased by an average of 6.2% for GFBLUP compared to GBLUP. This result indicated that inappropriate prior information was used in the GFBLUP method. One possible explanation is that the reference population size was not large enough to obtain accurate GWAS results.

The bias of genomic prediction of four traits in four populations, assessed with 20 replicates of fivefold CV, is presented in Table 2. The regression coefficients of WGBLUP were, on average, 0.929, 0.960, 0.892, and 0.900 for rainbow trout, sea bream, common carp, and Atlantic salmon, respectively, which produced slightly higher bias than GBLUP (0.992, 1.153, 1.046, and 1.019, respectively). However, a large bias was produced by the GFBLUP method for the four populations, as shown in Table 2. When the p values of the GWAS in GFBLUP were 0.05, the bias was on average 0.47 for the four traits in the four populations, indicating the poor performance of GFBLUP in this study.

### 3.4 Impact of low‐density SNP panels on genomic prediction

**FIGURE 1:**
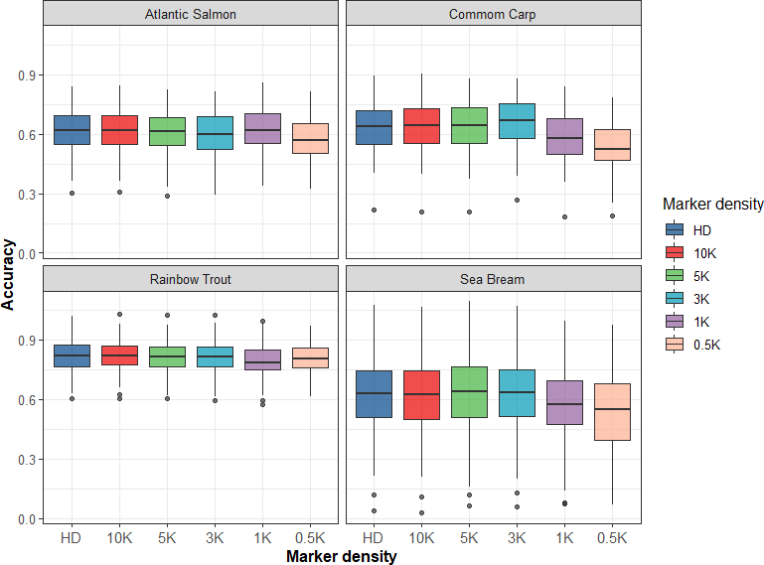
Accuracy of genomic prediction using GBLUP with different densities of SNP panels in four populations

**FIGURE 2:**
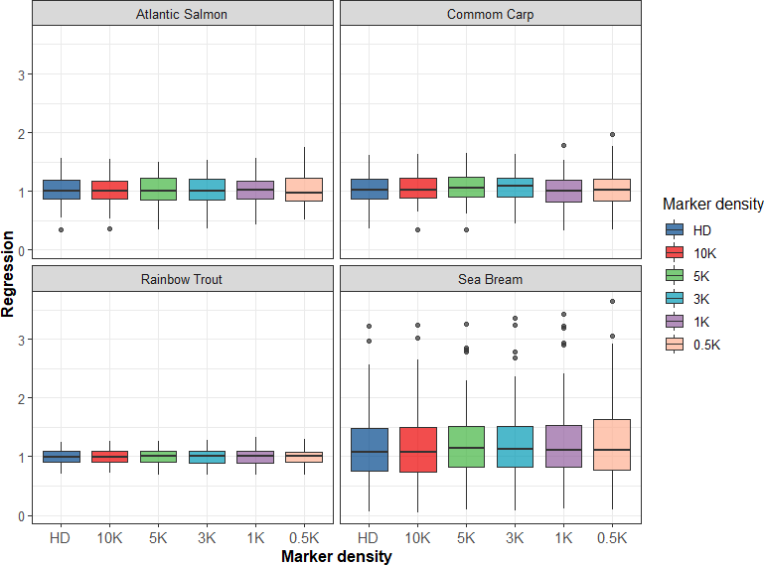
Regression coefficient of phenotypic values on GEBV using GBLUP with different densities of SNP panels in four populations

Since genotyping with medium‐ or high‐density SNP arrays is relatively expensive in aquaculture species, we evaluated the impact of low‐density SNP panel reduction based on LD on prediction accuracy for the four populations in this study. For GBLUP, five SNP panels with different densities (10K, 5K, 3K, 1K, and 0.5K) were used to assess prediction accuracy. In the four populations, the accuracy of genomic prediction tended to be stable with decreasing density of SNP panels from HD to 3K and then rapidly decreased, except that the prediction accuracy slightly increased when the SNP panel was 1K in the Atlantic salmon population, as shown in Figure 1. However, when the SNP panel was 0.5K, the accuracy of genomic prediction both decreased by 5.6%, compared with that using 3K and HD SNP panels. This means that when the density of the SNP panel is lower than 3K, high accuracy of genomic prediction cannot be maintained. Figure 2 presents the bias of genomic prediction. In general, the regression coefficients were close to 1 in the four populations under different SNP densities; although in some scenarios, bias slightly increased, for example, with the decrease of SNP density from HD to 0.5K, the regression coefficient increased from 1.153 to 1.232 in sea bream population. As a trade‐off between accuracy and bias, a relative SNP density of 3K was appropriate for obtaining high accuracy of GS for the four populations.

### 3.5 Impact of reference population size on genomic prediction

**FIGURE 3:**
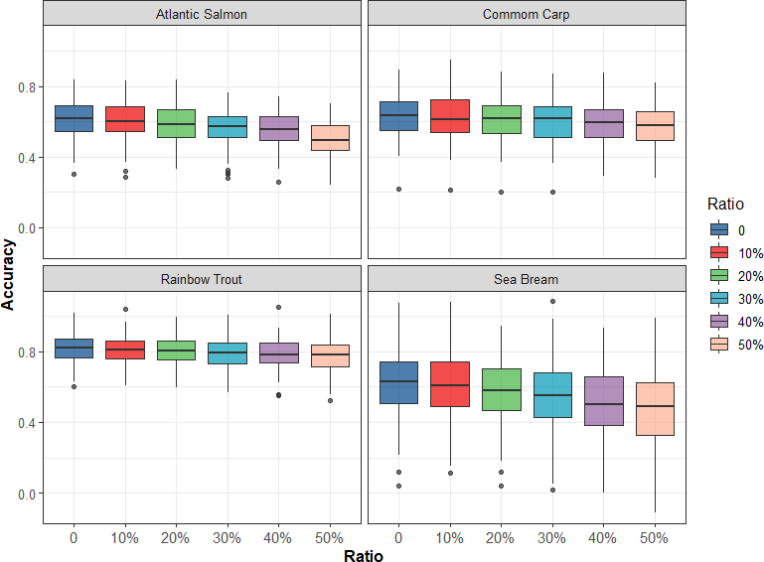
Accuracy of genomic prediction using GBLUP with different ratios of the reference population size decreased evenly from the full‐sib family

**FIGURE 4:**
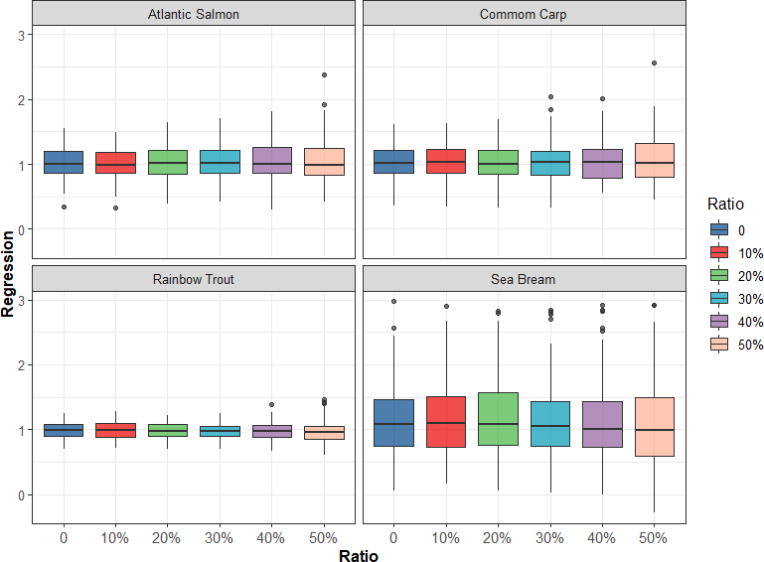
Regression coefficient of phenotypic values on GEBV using GBLUP with different ratios of the reference population size decreased evenly from the full‐sib family

To investigate additional cost‐effective ways of genotyping, reduced reference population sizes were tested by evenly sampling different ratios (10%–50%) of the reference population from each full‐sib family to assess the accuracy of genomic prediction. As shown in Figure 3, as the ratio of the reference population size increased, the accuracy of genomic prediction decreased for the four traits in the four populations. However, the accuracy of genomic prediction decreased at different rates in different populations. The accuracy of genomic prediction decreased by 3.6% and 5.6% when the reference population size decreased from 10% to 50% in rainbow trout and common carp populations, while the accuracy of genomic prediction decreased by 13% and 9.8% in sea bream and Atlantic salmon populations. In addition, in these four populations, when the reference population size was reduced by 10%, the accuracy of genomic prediction was almost unchanged, which means that genotyping for fewer individuals can achieve high prediction accuracy. Figure 4 presents the bias of genomic prediction from 20 replicates of fivefold CV in four fish populations by applying GBLUP with different ratios of the reduced reference population size; in general, the regression coefficients were close to 1 in the four populations with the reference population size decreased, indicating that the bias of genomic prediction was small in all situations.

### 3.6 Cost evaluation

In scenario (1), where all animals were genotyped with an HD panel, the total cost of genotyping was estimated to be $88860, $72840, $46620, and $44940 for Atlantic salmon, common carp, sea bream, and rainbow trout populations, respectively (see Table 3). However, in scenario (2), where all animals were genotyped with a 3K panel, the cost was estimated to reduce by 50% in four populations, with virtually no loss of genomic prediction accuracy for the traits measured. Additionally, in scenario (3), where all animals were genotyped with an HD panel and the reference population size was reduced by 10%, cost reduction was only 8% in the four populations. However, in scenario (4), where all animals were genotyped with a 3K panel and the reference population size was reduced by 10%, cost reduction was up to 54% in the four populations. This procedure greatly reduced the cost of GS, although it slightly reduced the accuracy of predicting GEBV in scenarios (3) and (4) (Figure S1).

**TABLE 3:** Genotyping cost (US$) using different genotyping strategies for four aquaculture populations

Scenarios^a^	Atlantic salmon	Common carp	Sea bream	Rainbow trout
(1) HD	88,860	72,840	46,620	44,940
(2) 3K	44,430	36,420	23,310	22,470
(3) HD, −10%	81,,750	67,008	42,888	41,340
(4) 3K, −10%	40,875	33,504	21,444	20,670

^a^ (1) HD = scenario (1): all animals were genotyped with a high‐density (HD) panel. (2) 3K = scenario (2): all animals were genotyped with a 3K panel. (3) HD, −10% = scenario (3): all animals were genotyped with an HD panel, and the reference population size was reduced by 10%. (4) 3K, −10% = scenario (4): All animals were genotyped with a 3K panel, and the reference population size was reduced by 10%.

## 4 DISCUSSION

In this study, we investigated the accuracy of genomic prediction of four traits in four aquaculture species. Our results revealed that the methods with marker information generally provided higher accuracies of genomic prediction than the traditional BLUP method; for example, GBLUP yielded 16.3% and 10.5% higher accuracies than BLUP in sea bream and Atlantic salmon populations, respectively. This suggests the advantage of GS in the breeding of aquaculture species. Similar results were reported by Houston et al. (2020), who reviewed several cases in which the accuracy of genomic prediction was higher than the traditional prediction method based on pedigree information in aquaculture species; on average, the prediction accuracy of disease resistance traits increased by 22%, and the prediction accuracy of growth‐related traits increased by 24%. The reason for this might be that the realized relationships among individuals are more accurately determined by marker information. In addition, BayesR assumes that the SNP effect follows four different normal distributions and produces a similar or higher prediction accuracy compared to GBLUP in this study (Table 2). Similar results have been reported for growth and reproduction traits in Yorkshire pigs (Song et al., 2017) and fatty acid composition traits in Korean Hanwoo cattle (Bhuiyan et al., 2018). Moreover, as reported from studies on real data (Hayes et al., 2009; Song et al., 2020; Zenger et al., 2019), the average prediction accuracies were not significantly different between the GBLUP and Bayesian methods, and the Bayesian method was computationally very demanding. The performance of Bayesian methods depends on their assumptions and the underlying genetic architecture of the traits.

In this study, two different methods, WGBLUP and GFBLUP, were used to improve genomic prediction accuracy in four aquaculture species. To date, the use of these two methods in aquaculture has rarely been investigated. The WGBLUP model uses a weighted genomic relationship matrix, which gives more weight to important markers. GBLUP assumes equal variance for all SNPs; this assumption is biologically incorrect but makes the statistics robust by limiting the number of unknown parameters (Meuwissen et al., 2001). To overcome the limitation of GBLUP, unequal weights for all SNPs were applied in WGBLUP, and the weights were calculated by an iterative procedure as described by Wang et al. (2012). In this study, GEBV was calculated based on three iterations for WGBLUP, as there was no difference between the results after the third and fourth iterations. WGBLUP produced higher prediction accuracies than GBLUP for all populations, as shown in Table 2. Many studies have also reported the advantages of WGBLUP over unweighted GBLUP (Gao et al., 2012; Tiezzi & Maltecca, 2015).

In addition, the strategy of weighted genomic relationship matrix is usually used in ssGBLUP (WssGBLUP); for example, Teissier et al. (2018) reported that the WssGBLUP methods were efficient for detecting SNPs associated with protein content and for a better prediction of genomic breeding values than ssGBLUP in French dairy goats, while similar accuracies were observed between WssGBLUP and ssGBLUP in Japanese flounder (Lu et al., 2020). However, it was not possible to apply WssGBLUP in this study, as no additional phenotypic individuals were available. Thus, if there are a large number of individuals without genotype but with phenotype information in the population, the application of WssGBLUP may further improve the accuracy of genomic prediction.

Moreover, the GFBUP with GWAS prior information was used to improve the accuracy of genomic prediction. Theoretically, GFBLUP has advantages over GBLUP, mainly because it allows the assignment of different weights to the genomic variants in the different genomic relationships based on their estimated genomic parameters, which can better fit the genetic architecture of the trait (Edwards et al., 2016; Fang et al., 2017). Fang et al. (2017) reported that the accuracy of genomic prediction was improved with GFBLUP compared to standard GBLUP in Holstein and Jersey cattle. Our previous study also reported that GFBLUP based on GWAS prior information could yield higher accuracy than GBLUP in a Yorkshire pig population (Song et al., 2019). However, the GFBLUP model with *p *= 0.05, when using GWAS prior information, yielded lower prediction accuracies and larger bias than GBLUP in this study. One possible reason is that the reference population size (1481, 1214, 749, and 777 in the four populations, respectively) was not large enough to obtain accurate GWAS results. A similar study was performed by Lu et al. (2020), in which the preselected 50K SNP‐based p‐value of GWAS did not increase the accuracy of genomic prediction. Another reason may be that only a few markers were selected with *p* values of 0.05, (582, 584, 618, and 1819 in four populations) to construct the genomic feature matrix in GFBLUP, which may be insufficient to construct an accurate genomic relationship matrix. In addition, different *p* values (0.1, 0.01) were set to select markers in the GWAS. However, similar results showed that GFBLUP had a lower prediction accuracy than GBLUP (results not shown).

Owing to the high fecundity of aquatic animals, it is necessary to genotype thousands of animals in each generation, which can be expensive. Therefore, to translate the benefits of GS into most aquaculture species, cost‐effective strategies need to be developed. Different strategies to choose SNPs for low‐density panels have been reported to reduce the costs of GS. For example, Robledo et al. (2018) evaluated the impact of reduced SNP density based on their minor allele frequency and their even position in the genome on prediction accuracy, and a reduction in marker density to ~2000 SNPs was sufficient to obtain high accuracy in Atlantic salmon. Kriaridou et al. (2020) investigated the accuracy of genomic prediction by randomly selecting SNP markers from SNP chips and found that SNP densities between 1000 and 2000 frequently result in selection accuracies that are very similar to those obtained with HD genotyping in four aquaculture species. The initial premise of GS is that each QTL is in LD with at least one SNP; SNPs that are distributed across the whole genome can explain most of the genetic variance (Meuwissen et al., 2001). However, when two SNPs are in high LD, their genotypic information is redundant, and only one is necessary to represent the variation in neighboring regions. Thus, in this study, to reduce the costs of GS, SNP panel genotypes were pruned based on LD to reduce SNP density. Our results showed that when the density of SNP panels was reduced to 3K, which was sufficient to obtain accuracies similar to those obtained using the whole dataset for four species (Figure 1), the cost of GS was estimated to be 50% lower than that of all animals genotyped with the HD panel (Table 3). High accuracy with low marker density may reflect the low effective population size and genome long‐range LD in the four aquaculture species, which may increase the predictive ability of a sparse SNP marker set. In addition, due to the large full‐sib family of aquaculture species, the use of low‐density SNP markers can obtain high genomic prediction accuracy (Lillehammer et al., 2013). This means that the markers not only capture LD between markers and QTLs but also the genetic relationship between individuals in aquaculture species, as related fish also share marker alleles.

In GS, the relationship between the reference and validation populations should be maximized to improve the accuracy of genomic prediction (Goddard & Hayes, 2009). However, the relationship within the reference population also affects the accuracy, and the average relationship within the animals included in the reference population should be low (Pszczola et al., 2012). Thus, the reference dataset (or training dataset) should cover the entire population. In this study, when the reference population size was reduced by 10% evenly from the full‐sib family, the accuracy of genomic prediction was almost unchanged, and the cost reduction was 8% in the four populations (Table 3). Furthermore, accuracy and bias of genomic prediction using GBLUP with 3K SNP panel and different ratios of the reduced reference population size were obtained (Figures S1 and S2), and a similar trend was found, as shown in Figures 3 and 4, where the cost of GS was the lowest (Table 3). The reason could be that since related animals of the full‐sib family may partly explain the same part of variation, reducing the size of the full‐sib family does not affect the accuracy of genomic prediction. This will greatly reduce the cost of GS, although it results in a slight reduction in the accuracy of predicting GEBV.

In addition, several other strategies for reducing GS costs could be explored: (1) genotype imputation, imputing from low‐to‐high‐density SNP markers, whole‐genome sequence SNP markers, and HD SNP markers, could increase the accuracy of GS without increasing the cost (Tsairidou et al., 2020; Vallejo et al., 2017; Yoshida et al., 2018). In this study, missing genotypes were imputed using the current data, and if HD reference genotype data were obtained, imputing 1K or 0.5K with higher density markers might improve the accuracy of genomic prediction; (2) genotype‐by‐sequencing (GBS) technologies are also likely to help reduce costs in aquaculture. GBS technology is widely used in animal, plant, and aquatic animal genetic analyses because of its simple, time‐saving, and low‐cost operation (Chung et al., 2017; Elshire et al., 2011; Li & Wang, 2017). Vallejo et al. (2016) compared the prediction effect of RAD‐seq and SNP chips on bacterial cold‐water disease resistance in rainbow trout and found that although the marker density of the SNP chip was higher (about 40K SNP–10K RAD‐seq), the selection accuracy of the two technologies was similar. (3) Compared with GBS data, low‐coverage sequencing data can be distributed more evenly. These markers could be associated with more QTLs with small effects. Theoretically, low‐coverage sequencing may be beneficial for GS. Zhang et al. (2021) found that whole‐genome sequencing at an average depth of 0.5× has almost the same accuracy as that of 8× in large yellow croaker (*Larimichthys crocea*). However, the efficiency of these strategies in actual genomic prediction requires further investigation.

## 5 CONCLUSION

It is very important to explore how to improve the accuracy of genomic prediction and develop cost‐effective strategies to accelerate the application of GS in aquaculture species. Our results showed that the methods with marker information were more accurate than the method based only on pedigree. The WGBLUP method yielded higher genomic prediction accuracy than GBLUP, while the GFBLUP model with *p *= 0.05 when using GWAS prior information yielded lower prediction accuracies and larger bias than GBLUP in this study. In addition, reducing SNP density based on LD pruning of SNP arrays and reducing the size of the full‐sib family are effective strategies to reduce the cost of GS.

## CONFLICT OF INTEREST

The authors declare that they have no competing interests.

## Supporting information

Fig S1

Fig S2

## Data Availability

The genotype and phenotype data can be accessed at https://www.g3journal.org/content/8/4/1195.supplemental (Atlantic salmon), https://www.g3journal.org/content/6/11/3693.supplemental (sea bream), https://figshare.com/articles/dataset/Supplemental_Material_for_Palaiokostas_et_al_2018/6281561 (common carp), and https://figshare.com/articles/Untitled_Item/7725668 (rainbow trout).
