# Supplementary figures and images for: Strategies to improve the accuracy and reduce costs of genomic prediction in aquaculture species
Source: Evol Appl. 2021 Jul 17;15(4):578–90. doi: 10.1111/eva.13262 (PMC9046917; doi:10.1111/eva.13262)

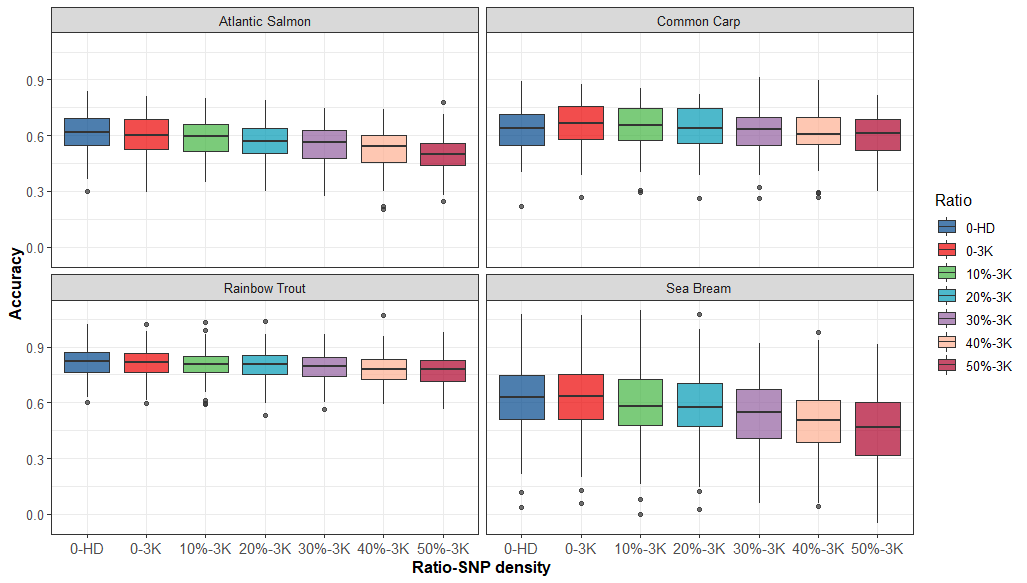

Supplement: Supplementary file 1 — Fig S1 [file EVA-15-578-s001.tiff]

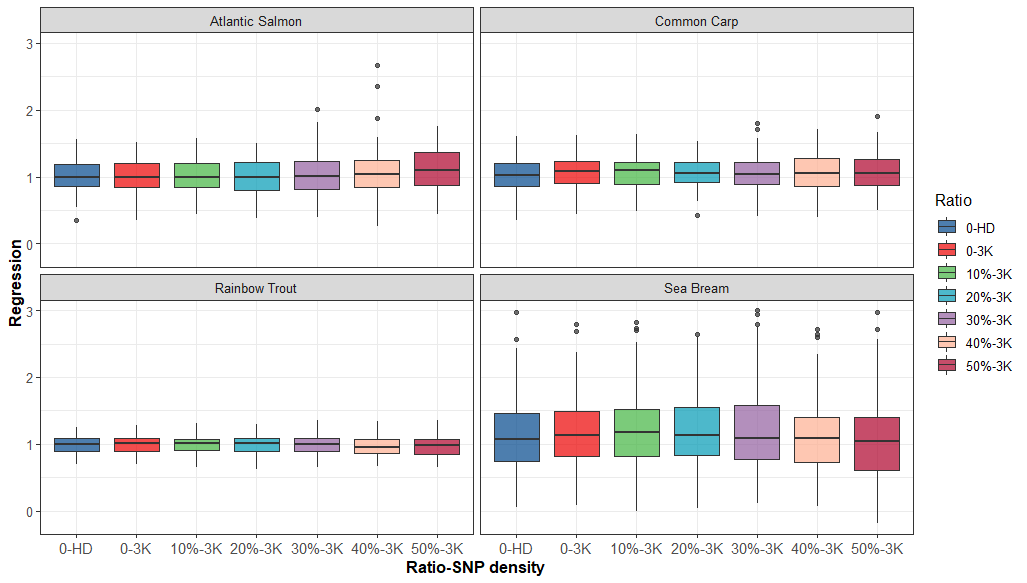

Supplement: Supplementary file 2 — Fig S2 [file EVA-15-578-s002.tiff]
